# Editorial: Recent advances in EEG (non-invasive) based BCI applications

**DOI:** 10.3389/fncom.2023.1151852

**Published:** 2023-03-02

**Authors:** Md. Kafiul Islam, Amir Rastegarnia

**Affiliations:** ^1^Department of Electrical and Electronic Engineering, Independent University, Dhaka, Bangladesh; ^2^Department of Electrical Engineering, Malayer University, Malayer, Iran

**Keywords:** editorial, EEG, brain computer interface (BCI), deep learning, artificial neural network, brain

## Background

Brain-Computer Interface (BCI) is a promising technique for establishing a direct link between the human brain and an external computerized device bypassing the normal pathway when it is not functional due to any brain/spinal cord related injury or diseases. BCI allows severely disabled persons to communicate with the outside world by controlling certain computerized devices such as a computer, wheelchair, neural prosthetics, etc. In addition to that, it is also used as a rehabilitation tool for stroke patients and people with similar needs. Another promising application of EEG-based BCIs is in the field of neurofeedback. This technique uses real-time EEG data to help individuals learn to control their own brain activity, which can be used to treat conditions such as depression, anxiety, and chronic pain.

Although there are different brain recording techniques, scalp EEG is the most popular among them in BCI research due to its mainly non-invasive nature with other attractive features such as fine temporal resolution, simple to use, portability and lower cost.

## Challenges

However, scalp EEG is very much prone to undesirable artifacts that come from the non-cerebral origin (Islam et al., 2016). The artifacts often severely contaminate the EEG recordings and modify the shape of a particular neurological event that drives the BCI system affecting the accuracy of the BCI performance (Islam et al., 2021). For example, artifacts can mistakenly cause an unintentional control of the BCI device. Therefore, handling of such offending artifacts is critical in BCI research for satisfactory performance and consequently, many techniques have been developed to get rid of artifacts that create misinterpretation in the signal analysis. Additionally, the current EEG recording technology is still far from being able to provide a natural and seamless communication channel between the brain and the computer due to its limited degree of freedom. Another challenge is that most of the signal processing and machine learning algorithms are not assessed for their suitability in online processing since real-time computation is a must for BCI applications. Another issue which is often not addressed: the feasibility and portability of a complete BCI system from the user's perspective.

Recent advances in signal processing techniques have now allowed researchers to design automatic artifact identification and removal algorithms to be able to implement online for real-world BCI applications. In addition, EEG based BCI systems have limited degree of freedom because of the non-invasive nature of EEG (lower signal resolution and mostly low frequency brain rhythms) compared to invasive brain recordings that allow recording single-neural activities or neural action potentials (neural spikes). Most BCI applications require wireless and lower number of recording channels for EEG headsets for easy mobility of users. This also limits the quality of the signals for converting to BCI commands. In addition, higher mobility of users will introduce so many motion artifacts due to subject/headset movements and thus makes it challenging to produce and process good quality EEG recordings for application in real-time BCI systems.

## Topic summary

The current Research Topic provides a useful overview of the capabilities and the applications of EEG based BCI: from advanced signal processing technique to remove facial muscle artifacts (Queiroz et al.) to the use of deep learning models to improve BCI performance (Jaipriya et al.); from the design of BCI experiments (Fisca et al.) to different BCI applications such as nighttime vehicle detection (Cui et al.), motor imagery BCI (Fujiwara et al.), SSVEP BCI (Israsena et al.); and finally two systematic reviews: one regarding the advances in deep learning for EEG-based BCI applications (Hossain et al.) and the other on brain-to-brain synchrony analysis to understand brain dynamics in non-invasive BCI applications (Nazneen et al.).

The first systematic review, Nazneen et al. critically assess the technological advancements in non-invasive brain-to-brain synchrony research to understand brain dynamics through neural connectivity analysis which is one of the future in BCI research. The second review, Hossain et al. systematically evaluates the last 5 years of work on deep learning models to compare their pros and cons and suitability in EEG-based BCI applications. This review has predicted that deep learning models have the required potential to lead the future of BCI research given that a large dataset is available.

The common motivation of two articles Queiroz et al. and Israsena et al. is the use of lower no. of EEG channels to make BCI systems wearable and portable. The former discusses facial muscle artifacts removal from single channel EEG to enhance signal quality while the latter proposes a CNN-Based Deep Learning model to enhance SSVEP BCI performance.

Fujiwara et al. proposes a deep residual CNN model for testing unknown subjects which eventually improves MI BCI performance. Fisca et al. investigates the design of BCI experiments on the biases of classifier models to different stimulus setups. A Masking Empirical Mode Decomposition (MEMD) based Feed Forward Back Propagation Neural Network (MEMD-FFBPNN) has been proposed by Jaipriya et al. to improve reliability and accuracy of the MI BCI system. Cui et. al. demonstrates a new BCI model based on Rapid serial visual presentation (RSVP) where the authors use Dynamic probability integration (DPI) technique to fusion both human and computer vision to improve nighttime vehicle detection performance.

In summary, all these eight articles in this Research Topic gave excellent insights into the current trends in EEG based BCI systems, their challenges and future potentials.

## Future direction

Eventually the performance of BCI applications largely depend on some sort of machine learning or deep learning models which means the better a model is trained with EEG data, the better the outcome of BCI performance. This dataset will largely depend on reliable and repeatable trials of BCI experiments. Therefore, one of the directions should focus on creating a sufficiently large database with reliable experiment setups and these datasets need to be made public in order for other researchers to use and train their models as much as possible.

Despite the challenges, the future of EEG-based BCIs looks bright. With continued research and development, we can expect to see even more powerful and sophisticated BCIs that can help people with disabilities, improve our understanding of the brain, and even augment human capabilities in various fields such as healthcare, entertainment, and education.

## Author contributions

All authors listed have made a substantial, direct, and intellectual contribution to the work and approved it for publication.
